# Perioperative Management of a Pediatric Patient With Koolen-de Vries Syndrome Presenting for Posterior Spinal Fusion

**DOI:** 10.14740/jmc5234

**Published:** 2026-01-04

**Authors:** Alaa Soliman, Jonathan Schmidt, Joseph D. Tobias, Ashley Smith

**Affiliations:** a Department of Anesthesiology & Pain Medicine, Nationwide Children’s Hospital, Columbus, OH, USA; b The Ohio State University College of Medicine, Columbus, OH, USA; c Department of Anesthesiology & Pain Medicine, The Ohio State University College of Medicine, Columbus, OH, USA

**Keywords:** Koolen-de Vries syndrome, Hypotonia, Intellectual disability, Facial dysmorphism, Pediatric anesthesiology

## Abstract

Koolen-de Vries syndrome (KdVS), caused by haplo-insufficiency of the *KANSL1* gene, is a rare neurodevelopmental disorder characterized by hypotonia, intellectual disability, facial dysmorphism, and multi-system end-organ involvement. Given the potential for skeletal and central nervous system involvement, patients with KdVS may require anesthetic care during diagnostic imaging or surgical procedures. Due to the rarity of the syndrome, information regarding anesthetic management remains sparse, derived primarily from isolated case reports. We present the anesthetic management of a 13-year-old patient with KdVS during posterior spinal fusion for neuromuscular scoliosis. Previous case reports are reviewed, the spectrum of end-organ involvement is presented, and options for perioperative care are discussed.

## Introduction

Koolen-de Vries syndrome (KdVS) is a rare, genetic (autosomal dominant) neurodevelopmental disorder caused by alterations in the genes at chromosome 17q21.31, which disrupt the KAT8 regulatory non-specific lethal complex subunit 1 (KANSL1) [1]. It can arise either from a recurrent microdeletion or protein truncating variant within the *KANSL1* gene. This gene encodes a protein that regulates gene expression via chromatin modification, therefore influencing the development and function of multiple organ systems [2]. It was first described in 2006, at which time it was identified as primarily due to haplo-insufficiency of the *KANSL1* gene. Clinical manifestations of KdVS commonly include hypotonia, global developmental delay, moderate intellectual disability, seizures, structural central nervous system (CNS) anomalies, and distinctive craniofacial features (facial dysmorphism).

Typical craniofacial features of KdVS include a long face with an open mouth, a tubular or pear-shaped nose with a bulbous tip, and relative macrocephaly with a broad forehead. Ocular manifestations include up-slanting or narrow palpebral fissures, ptosis, and epicanthal folds. Other findings include a high-arched palate, everted lower lip, broad chin, and prominent ears [3]. Additional phenotypic and end-organ involvement may include musculoskeletal deformities including neuromuscular scoliosis, epilepsy, congenital cardiac defects, sociable behavioral profile, urogenital malformations, and occasionally hearing loss or ophthalmic involvement [4-6]. Given the defined end-organ involvement, patients with KdVS frequently require anesthetic care for both diagnostic procedures and surgical interventions. Due to the rarity of the syndrome, anesthetic management guidance remains sparse, derived primarily from isolated case reports [7, 8]. We outline the anesthetic management of a 13-year-old adolescent with a confirmed diagnosis of KdVS undergoing posterior spinal fusion (PSF) surgery for neuromuscular scoliosis. Previous case reports of anesthetic care for patients with KdVS are reviewed, the spectrum of end-organ involvement presented, and options for perioperative care discussed.

## Case Report

This case was reviewed and presented in accordance with the Institutional Review Board guidelines of Nationwide Children’s Hospital (Columbus, Ohio). Written, informed consent was obtained for anesthetic care and the use of de-identified information for publication.

A 13-year-old, 46.5 kg adolescent with KdVS and neuromuscular scoliosis presented for PFS with multilevel segmental instrumentation, thoracic and lumbar osteotomies, and bone grafting. KdVS diagnosis was confirmed with microarray analysis 2 months after delivery (microdeletion syndrome in chromosome 17q21.31 associated with a deletion in the *MAPT* gene). The patient’s complex past medical history included Lennox-Gastaut syndrome with intractable epilepsy, moderate intellectual disability, hypotonia, developmental delay, short stature, scoliosis, Hashimoto’s thyroiditis with hypothyroidism, laryngomalacia, congenital nystagmus with esotropia and astigmatism, facial palsy, eustachian tube dysfunction, conductive hearing loss, gastroesophageal reflux disease, gastrostomy-tube dependence, mild intermittent asthma, and a history of hypertension with a patent foramen ovale and muscular ventricular septal defect (VSD). The patient was non-verbal and dependent on a modified ketogenic diet administered via gastrostomy tube for seizure management. Current medications included levothyroxine (112 µg daily), clobazam (10 mg daily), levetiracetam (750 mg twice daily), norethindrone acetate via G-tube, potassium bicarbonate-citric acid (25 mEq twice daily to prevent metabolic acidosis with the ketogenic diet), and as-needed intranasal midazolam intranasal for breakthrough seizures. The patient had multiple previous surgeries including esophagogastroduodenoscopy with percutaneous gastrostomy tube placement, nasal turbinate reduction, tonsillectomy with adenoidectomy, magnetic resonance imaging (MRI) of the brain and spine, laryngoscopy and bronchoscopy, tympanostomy tube placements, and eye surgery. There was a prior history of respiratory difficulties following a previous anesthetic. The event occurred when the patient was 7 years old, undergoing tonsillectomy with revision adenoidectomy. During emergence from anesthesia, following tracheal extubation, the patient developed upper airway obstruction with stridor and oxygen desaturation. Treatment included placement of an oral airway and use of continuous positive airway pressure (CPAP) via an anesthesia bag and mask. He was eventually weaned to room air and admitted postoperatively to the inpatient ward.

On pre-anesthetic evaluation, the blood pressure was 139/101 mm Hg, but the other vitals were stable. Physical examination revealed a normal cardiac and respiratory exam. The airway examination revealed small mouth opening (1 - 2 finger breadths) and a short thyromental distance of less than 3 finger breadths. Echocardiography was unremarkable as the previous muscular VSD and patent foramen ovale had spontaneously closed. Thyroid function tests were within normal limits as were the hemoglobin, hematocrit, and the coagulation profile. The patient was assigned an American Society of Anesthesiologists (ASA) physical status classification IV due to associated severe end-organ involvement including upper airway concerns.

The patient was held *nil per os* (NPO) for 6 h. Premedication included oral midazolam for anxiolysis and aprepitant per institutional protocol for prevention of postoperative nausea and vomiting. The difficult airway cart along with indirect laryngoscopy was available in the operating room. The patient was transported to the operating room, routine ASA monitors were applied, and anesthesia was induced by the inhalation sevoflurane in 100% oxygen via facemask followed by the insertion of a peripheral, 18-gauge, intravenous cannula. Propofol (2 mg/kg) and fentanyl (2 µg/kg) were administered to deepen the level of anesthesia and once adequate bag-valve-mask ventilation was demonstrated, rocuronium (0.6 mg/kg) was administered to facilitate endotracheal intubation. Airway management was achieved using an indirect video laryngoscopy (GlideScope^®^ LowPro S3 blade), which provided a grade I view of the glottis. Endotracheal intubation was successful on the first attempt using a 5.5 mm cuffed endotracheal tube (ETT) with a stylet. Ultrasonography was used for placement of a second peripheral intravenous cannula and a radial arterial cannula. Mechanical ventilation was achieved with volume-guaranteed, pressure-controlled ventilation with a tidal volume of 8 mL/kg and with the rate being adjusted to maintain the end-tidal CO_2_ at 30 - 40 mm Hg. The patient was positioned prone on Jackson table and pressure points were padded. General anesthesia was maintained with desflurane (1.5-2.5%) in 50% oxygen/air, remimazolam (5 - 20 µg/kg/min), methadone (0.1 mg/kg), remifentanil (0.1 - 0.3 µg/kg/min), and lidocaine (1 mg/kg/h) [9, 10]. Techniques to decrease the need for allogeneic transfusions included tranexamic acid (50 mg/kg followed by 5 mg/kg/h) and intraoperative cell salvage. Surgical site infection prophylaxis was provided with cefazolin (2 g) administered at 3-h intervals. Maintenance of normothermia was achieved by warming the operating room, applying a forced-air warming blanket, and warming intravenous fluids. The duration of the surgery was 8.5 h. Total blood loss was 200 mL and urine output was 320 mL. Intraoperative fluid management consisted of 750 mL of 5% albumin, one unit of packed red blood cells, and 3,070 mL of isotonic fluids (Normosol-R). Prophylaxis for postoperative nausea and vomiting was provided by intraoperative dexamethasone (4 mg) and ondansetron (4 mg). After completion of the surgery, sugammadex (200 mg) was administered to ensure reversal of residual neuromuscular blockade, the patient was turned supine, and tracheal extubation was performed after the patient regained baseline consciousness. Postoperative analgesia was provided by fixed interval dosing of acetaminophen and ketorolac plus morphine administered via nurse-controlled analgesia. The patient was transported to the post-anesthesia care unit and then discharged to the inpatient ward. All routine home medications were resumed that evening. Refeeding with the home ketogenic diet regimen was started on postoperative day (POD) 1 and the patient was discharged home on POD 4.

## Discussion

KdVS is an autosomal dominant neurodevelopmental disorder, resulting from alterations in the genes on the long arm of chromosome 17. Involvement of the genes at this locus results in various end-organ clinical manifestations including craniofacial involvement with distinctive facial dysmorphism and primarily CNS effects (hypotonia, global developmental delay, moderate intellectual disability, seizures, and structural CNS anomalies). Additional involvement may include musculoskeletal deformities (neuromuscular scoliosis as noted in our patient), congenital cardiac defects, urogenital malformations, and occasionally hearing loss or ophthalmic involvement. Of primary concern during perioperative care is the potential for difficulties with airway management (bag-valve-mask ventilation, upper airway obstruction, or difficulties with endotracheal intubation) related to craniofacial involvement and dysmorphism (Table 1). Given the rarity of the syndrome, published experience with the perioperative management of patients with KdVS remains limited, with only two prior reports describing anesthetic care in children with this condition (Table 2) [7, 8].

**Table 1:** Perioperative Concerns of Anesthetic Care in Patients With Koolen-de Vries Syndrome

1. Craniofacial dysmorphism: a) Difficulties with bag-valve-mask ventilation or endotracheal intubation; b) Upper airway obstruction during anesthetic induction or recovery phase.
2. Upper airway involvement: a) Laryngomalacia; b) Tracheomalacia.
3. Central nervous system involvement: a) Hypotonia; b) Global developmental delay with moderate intellectual disability; c) Seizures; d) Structural central nervous system abnormalities.
4. Congenital heart disease: a) Atrial septal or ventricular septal defects; b) Patent ductus arteriosus; c) Patent foramen ovale; d) Aortic valve involvement (hypoplastic or bicuspid valve).
5. Aspiration risk due to pharyngeal dyscoordination and gastro-esophageal reflux
6. Urogenital malformations
7. Musculoskeletal involvement including neuromuscular scoliosis and joint contractures: a) Positioning challenges during anesthetic care; b) Difficult vascular access.
8. Hearing loss
9. Ophthalmic involvement

**Table 2:** Previous Reports of Anesthetic Care in Patients With Koolen-de Vries Syndrome

Author and reference	Patient demographic	Anesthetic technique	Comments and outcome
Kavakli [7]	A 2-year-old, 11.6 kg girl requiring sedation for sedation for MRI. Echocardiography revealed patent foramen ovale with trivial tricuspid regurgitation. Facial dysmorphism: tubular or pear-shaped nose, bulbous nasal tip, epicanthal folds, up-slanting palpebral fissures, and telecanthus.	Premedication with oral midazolam (0.5 mg/kg) administered 30 min before the procedure, followed by placement of an intravenous cannula. Procedural sedation included intravenous propofol (0.5 mg/kg) and ketamine (1 mg/kg).	Airway approach: Spontaneous ventilation with a native airway during the procedure. Nasal cannula oxygen at 1 L/min. No intraoperative concerns. Patient discharged home the same day.
Zhao and Zuo [8]	A 21-month-old, 11 kg boy for orchiopexy. History of associated laryngo-tracheomalacia admitted for orchiopexy. Echocardiography revealed a patent foramen ovale. Facial dysmorphism: long face, broad forehead, blepharophimosis, ptosis, epicanthal folds, tubular pear-shaped nose, and large prominent ears.	Intravenous induction with midazolam (0.75 mg), fentanyl (20 µg), and etomidate (6 mg). Maintenance anesthesia with sevoflurane (1-3%) and remifentanil infusion (0.1 - 0.15 µg/kg/min). Postoperative analgesia with a caudal epidural block (12 mL of 0.25% ropivacaine).	Airway approach: A supraglottic airway (i-gel) was placed, but failed to provide an adequate airway as inspiratory pressures with high with low tidal volumes. Therefore, an ETT was placed (no mention made of technique or difficulty with placement), and the case was completed. Airway events: Following tracheal extubation, agitation, upper airway obstruction, and oxygen desaturation were noted. Treatment included lateral positioning and the administration of a dose of etomidate which resulted in some improvement. Intermittent upper airway obstruction and stridor were noted for the first 48 postoperative hours.

ETT: endotracheal tube; MRI: magnetic resonance imaging.

These two case reports demonstrate the usual associated organ system involvement of patients with KdVS including craniofacial dysmorphism, generalized hypotonia, moderate intellectual disability, and multisystem involvement that can make airway management challenging. The report of Kavakli outlined procedural sedation during MRI in a 2-year-old, 11.6-kg child [7]. Sedation was provided by a combination of intravenous propofol (0.5 mg/kg) and ketamine (1 mg/kg) during the 20-min procedure. Supplemental oxygen was provided at 1 L/min via nasal cannula. The MRI scan was completed uneventfully and following the procedure, the patient was observed in the recovery room for 60 min before discharge. A more challenging case was presented by Zhao and Zuo who shared their experience of providing anesthetic care for a 21-month-old, 11 kg boy with KdVS during orchiopexy. Comorbid conditions included tracheo/laryngomalacia and a patent foramen ovale [8]. The patient’s history was remarkable for previous airway issues including a propensity to develop upper airway infections as well as stridor with these infections. Given the potential for a difficult airway, anesthesia was induced with the inhalation of incremental concentrations of sevoflurane. During anesthetic induction, upper airway obstruction was noted which resolved with the application of CPAP. Airway examination with indirect video-laryngoscopy (Glidescope^®^) revealed that the epiglottis was long, curved, and swung with each breath. A supraglottic airway (i-gel) was placed but failed to provide adequate airway coverage, requiring high inspiratory pressures with low tidal volumes. Therefore, an ETT was placed (no mention made of technique or difficulty with placement), and the case was completed. Following tracheal extubation, agitation, upper airway obstruction, and oxygen desaturation were noted, which were treated with lateral positioning and the administration of a dose of etomidate. Intermittent upper airway obstruction and stridor persisted for the first 48 postoperative hours.

As with all anesthetic care, the first step involves a focused history and physical examination with identification of end-organ involvement. Patients with known genetic syndromes pose a variety of challenges to the anesthesia provider including the potential for difficulties with airway management, bag-valve-mask ventilation or endotracheal intubation [10, 11]. This is a concern in patients with KdVS syndrome, as facial dysmorphism is a hallmark phenotypic presentation with distinctive craniofacial features including a long face, broad forehead, narrow high arched palate, micrognathia, and limited mouth opening that may complicate bag-valve-mask ventilation and direct laryngoscopy [6]. The preoperative airway evaluation in our patient was limited by cognitive impairment. Examination revealed micrognathia, small mouth opening (1 - 2 finger breadths), and a short thyromental distance (< 3 finger breadths). We chose to maintain spontaneous ventilation and not to provide neuromuscular blockade until adequate bag-valve-mask ventilation was demonstrated. Additionally, the equipment required for dealing with the difficult airway, including indirect video laryngoscopy and the difficult airway cart, was in the room during anesthetic induction [12, 13].

Additional airway concerns which may impact postoperative respiratory function include laryngomalacia or tracheomalacia as well as poor upper airway control related to hypotonia or CNS involvement. Airway involvement, either functional or anatomical including laryngo-tracheomalacia, may affect approximately 20% of patients with KdVS [5, 6]. This, combined with the residual effects of anesthetic agents and poor airway control, may increase the risk of airway compromise, including upper airway obstruction, during or following general anesthesia or sedation. Whenever feasible, short-acting anesthetic agents such as desflurane and remifentanil are recommended to avoid the impact of residual anesthetic effects on postoperative respiratory function. As noted in the report of Zhao and Zuo, postoperative respiratory concerns may arise related to intermittent upper airway obstruction. These were treated with CPAP during the induction of anesthesia and positioning with attention to chest physiotherapy and airway clearance postoperatively. Non-invasive respiratory support (bilevel positive airway pressure (BiPAP) support) may also be used to prevent the need for reintubation due to respiratory insufficiency or failure when upper airway concerns progress [14]. As indicated, close monitoring of postoperative respiratory function may require intensive care unit (ICU) admission.

An additional concern that may impact airway management is the potential risk of aspiration given pharyngeal dyscoordination and gastro-esophageal reflux, which may have a higher incidence in patients with cognitive impairment [15]. When severe, these concerns may warrant the use of intravenous induction and even rapid sequence intubation (RSI). Given the confirmed NPO status of our patient, the limited clinical concerns of significant aspiration, and the potential for difficulties with airway management, inhalation induction with the maintenance of spontaneous ventilation was chosen.

Congenital cardiac anomalies are relatively common in patients with KdVS, reported in up to 25-50% of patients [16]. Congenital heart anomalies most commonly include atrial septal defect or VSD. Other reported cardiac anomalies have included patent ductus arteriosus, patent foramen ovale, aortic valve involvement (hypoplastic or bicuspid valve), common left pulmonary vein, dysplastic pulmonary valve, anomalous right subclavian artery, doubly committed VSD, and mitral insufficiency. Our patient had a previously documented muscular VSD and a patent foramen ovale, both of which had closed spontaneously by the time of the current surgery. Given the association with congenital heart disease, preoperative echocardiography is recommended. Of note, our patient also had a history of hypertension, which has not been a commonly reported comorbid condition associated with KdVS. Our patients’ blood pressure at her pre-anesthetic evaluation was 139/101 mm Hg, a value consistent with previous readings. Routine screening is recommended during the perioperative period to identify patients with pre-existing hypertension which may require perioperative control prior to elective procedures [17].

Hypotonia is a core feature of KdVS and may impact perioperative care including choice and administration of neuromuscular blocking agents (NMBAs). Given the paucity of case reports regarding anesthetic care in patients with KdVS, there is limited evidence-based medicine regarding the choice of NMBA. Theoretical concerns extrapolated from general use of NMBAs in patients with hypotonia and skeletal muscle issues suggest avoidance of succinylcholine, given the potential for rhabdomyolysis and hyperkalemia [18]. Although non-depolarizing NMBAs may be used, their response and duration may be exaggerated in patients with hypotonia and neuromuscular involvement [19, 20]. For our patient, a single dose of rocuronium was administered to facilitate endotracheal intubation. Spontaneous recovery occurred within 30 - 60 min and allowed for effective neurophysiological monitoring (motor-evoked potentials). When repeated doses of NMBAs are administered, train-of-four monitoring may be indicated to guide dosing and documented full reversal. Reversal of residual neuromuscular blockade with sugammadex may provide an additional margin of safety and ensure full reversal in patients with hypotonia or neuromuscular disorders [21].

CNS involvement includes cognitive impairment, seizures, and structural anomalies and may be observed in up to 80% of patients with KdVS. Structural brain abnormalities identified on routine imaging may include ventriculomegaly, aplasia or hypoplasia of the corpus callosum, hydrocephalus, Chiari 2 malformation, intraventricular hemorrhage, and an ovoid hippocampus [15]. As noted in our patient, seizure disorders are a frequent comorbid condition that may impact perioperative care. Patients should continue their routine anti-seizure medications including on the morning of surgery, regardless of NPO status [22]. Specific perioperative concerns regarding maintenance of the ketogenic diet during perioperative care may need to be considered [23]. Although it has been postulated that specific agents may activate the electroencephalogram (EEG) and hence augment seizure activity, in general, the inhalational and intravenous anesthetic agents are anticonvulsants and there is limited need to adjust choice of anesthetic agents in patients with an underlying seizure disorder [24]. However, given the physiologic stress of surgery, close monitoring during the postoperative period is required to identify break-through seizures. Most individuals with KdVS are sociable and have an amiable affect; however, behavioral problems have been noted to include autism, hyperactivity, shyness, anxiety, phobias, impulsive and stereotypic behavior, psychosis, and depression [25]. Endocrine involvement in KdVS is unusual aside from anecdotal reports. Concerns have included growth hormone deficiency with associated pituitary structural abnormalities and central hypothyroidism. In the case reported by Zhao and Zuo, the patient was noted to have hypothyroidism. This remains uncertain whether such coexisting conditions are directly associated with KdVS or merely unrelated comorbid conditions.

Musculoskeletal abnormalities, particularly scoliosis, are commonly observed in individuals with KdVS with a reported prevalence of 56% [26]. Progressive scoliosis can contribute to restrictive lung disease, and when combined with hypotonia, have an added impact on perioperative upper airway control and respiratory function, predisposing these patients to respiratory insufficiency. Reported cases highlight that scoliosis in KdVS can progress rapidly, sometimes necessitating orthopedic surgical intervention as noted in our patient. Joint and soft tissue contractures or orthopedic deformities may impact vascular access especially during major surgical interventions. Our clinical practice supports the utility of ultrasound in guiding placement of venous and arterial cannulas. Contractures may also impact intraoperative positioning, especially in the prone position on the Jackson table. Padding of bony prominences is imperative to prevent skin breakdown during prolonged procedures or immobilization. Growth failure, nutritional issues, and CNS disabilities may predispose to hypothermia. Intraoperative care includes continuous temperature monitoring and the use of overhead heating lights, forced air warming devices, and increased room temperature.

### Learning points

In summary, we present the anesthetic management of a 13-year-old adolescent with KdVS, a rare neurodevelopmental disorder, during PSF for neuromuscular scoliosis. Perioperative care should include a thorough preoperative evaluation, multidisciplinary consultation as needed, and individualized anesthetic management with postoperative monitoring. Of primary concern during anesthetic care is the potential for difficulties with bag-valve-mask ventilation and endotracheal intubation. Additional primary anesthetic concerns include cognitive impairment, seizure disorders, hypotonia which may impact choice of NMBA, and the potential for postoperative respiratory failure related to hypotonia, poor airway control, and pulmonary aspiration. Given the severe associated CNS involvement, short-acting anesthetic agents (desflurane, remifentanil) may be preferred to facilitate emergence from anesthesia.

## Data Availability

The data supporting the findings of this case report are available from the corresponding author upon reasonable request.
